# Should samples be weighted to decrease selection bias in online surveys during the COVID-19 pandemic? Data from seven datasets

**DOI:** 10.1186/s12874-022-01547-3

**Published:** 2022-03-06

**Authors:** Chadia Haddad, Hala Sacre, Rony M. Zeenny, Aline Hajj, Marwan Akel, Katia Iskandar, Pascale Salameh

**Affiliations:** 1 INSPECT-LB (Institut National de Santé Publique, d’Épidémiologie Clinique et de Toxicologie-Liban), Beirut, Lebanon; 2 Research Department, Psychiatric Hospital of the Cross, P.O. Box 60096, Jal Eddib, Lebanon; 3 Department of Pharmacy, American University of Beirut Medical Center, Beirut, Lebanon; 4 Laboratoire de Pharmacologie, Pharmacie Clinique Et Contrôle de Qualité Des Médicament, Saint-Joseph University, Beirut, Lebanon; 5 Faculty of Pharmacy, Saint-Joseph University, Beirut, Lebanon; 6 School of Pharmacy, Lebanese International University, Beirut, Lebanon; 7 Faculty of Pharmacy, Lebanese University, Beirut, Lebanon; 8 UMR1295, INSERM, Université Toulouse III Paul-Sabatier, Toulouse, France; 9 University of Nicosia Medical School, Nicosia, Cyprus; 10 School of Medicine, Lebanese American University, Byblos, Lebanon

**Keywords:** Weighting, Online surveys, Relative difference, Bias, COVID-19, Pandemic

## Abstract

**Background:**

Online surveys have triggered a heated debate regarding their scientific validity. Many authors have adopted weighting methods to enhance the quality of online survey findings, while others did not find an advantage for this method. This work aims to compare weighted and unweighted association measures after adjustment over potential confounding, taking into account dataset properties such as the initial gap between the population and the selected sample, the sample size, and the variable types.

**Methods:**

This study assessed seven datasets collected between 2019 and 2021 during the COVID-19 pandemic through online cross-sectional surveys using the snowball sampling technique. Weighting methods were applied to adjust the online sample over sociodemographic features of the target population.

**Results:**

Despite varying age and gender gaps between weighted and unweighted samples, strong similarities were found for dependent and independent variables. When applied on the same datasets, the regression analysis results showed a high relative difference between methods for some variables, while a low difference was found for others. In terms of absolute impact, the highest impact on the association measure was related to the sample size, followed by the age gap, the gender gap, and finally, the significance of the association between weighted age and the dependent variable.

**Conclusion:**

The results of this analysis of online surveys indicate that weighting methods should be used cautiously, as weighting did not affect the results in some databases, while it did in others. Further research is necessary to define situations in which weighting would be beneficial.

**Supplementary Information:**

The online version contains supplementary material available at 10.1186/s12874-022-01547-3.

## Background

Generally used for marketing purposes, online surveys have recently become a popular data-gathering tool in scientific research [1], mainly helpful during the COVID-19 pandemic. Besides protecting data collectors from infection, cost savings, simplicity of data collection, ease of processing findings, flexibility in questionnaire design, and the ability to contact respondents across national borders are all arguments in its favor [1]. However, the use of web surveys has triggered a heated debate regarding their scientific validity [2, 3].

The main argument against web surveys is the selection bias of the sample, which is not chosen at random, the target population being a convenience sample rather than a probability sample [1]. This non-probability method of selection is generally problematic, leading to an unequal probability of selection. Bias further occurs since specific characteristics (such as age, education, gender) are under- or over-represented in the gathered sample, thus impacting the reliability of the results [1]. Even a well-designed sampling plan would frequently result in the survey being completed by too many women and not enough men or by too many young people and not enough elderly individuals. Furthermore, all these factors might be linked to different health-related variables, attitudes, and behaviors that survey researchers are interested in [4].

Selection bias occurs in studies that use online surveys as it only reaches a subgroup of the target population [5]. Only literate people, those who have access to the internet, and those sufficiently interested in the topic can complete online surveys [5]. For example, when a subgroup is targeted (thus overrepresented, such as literate people or those with access to the internet), selection bias will generally increase as the target population becomes less diverse, resulting in biased findings [5, 6]. Sometimes, a survey about COVID-19 would only attract a specific subgroup of people interested in the topic. However, during infectious disease outbreaks, a quick online survey is necessary to reach a large number of people in a short time to collect the needed information [7]. Moreover, various types of problems and errors are encountered in the data collected online (information bias), leading to concerns about the quality and reliability of the resulting scientific information [1].

To overcome biases and improve the quality of online survey findings, many authors have adopted weighting methods [1, 4], such as rectifying imbalances between the survey sample and the population by applying these methods to adjust demographic characteristics (gender, age, ethnicity, educational background, and geographic area) [4]. Because some factors of interest may not always have a strong enough link with demographic weighting variables, weighting methods can only compensate for proportionality, not always representativeness [8]. Hence, the considerable debate about weighting methods and their effect on variance during analysis, as some researchers claim that weighting has little potential for eliminating biases in web surveys [9]. As variance is used to calculate confidence intervals and hypothesis tests, weighting data would raise the variance of estimates [10], leading to a loss of accuracy [10]. Nevertheless, researchers are often willing to accept inaccuracy to obtain unbiased estimates [10].

A direct comparison of unweighted and weighted samples has rarely been performed in the literature [11, 12]. From a practical perspective, comparing the two techniques is critical because they may provide different findings of the overall impact strength, outcome consistency across studies, and other variables’ effect on the association.

Two studies comparing weighted and unweighted estimates from online samples have revealed that demographic weighting decreased bias in some situations while it substantially increased it in others [11, 12]. Recent research using aggregated data to evaluate racial/ethnic inequities in COVID-19 mortality has found that weighted population distributions underestimated the excess burden of COVID-19 among African American and Latin individuals, compared with analyses conducted with an unweighted population [13].

Consequently, this work aims to compare weighted and unweighted association measures after adjustment over potential confounding, taking into account dataset properties such as the initial gap between the population and the selected sample, the sample size, and the variable types.

## Methods

### Databases

This study assessed seven datasets of different sample sizes collected by our team between 2019 and 2021 during the COVID-19 pandemic through online cross-sectional surveys using the snowball sampling technique. All seven datasets consisted of basic demographic variables (including age and gender), major independent variables, and different outcome variables.

### Procedure

Identical questions measuring basic demographics were used in each database. Weighting techniques were applied and mostly accounted for sociodemographic differences between the online sample and the target population.

The formula of such weights [14] was: w_*i*_ = *p*_*p*_*/p*_*s*_, where *p*_*p*_ is the population proportion, and *p*_*s*_ is the (web) sample proportion.

In each database, a major outcome variable associated with the demographic variables was chosen, in addition to an independent variable. Weighted versus unweighted results were compared in all datasets. Details about each dataset are presented in Table 1.

**Table 1:** Description of the seven datasets used

	**Sample size**	**Dependent variable (s)**	**Independent variable (s)**
Dataset 1	310	Practice toward COVID-19	Attitude toward COVID-19
Dataset 2	509	Stress (The Beirut Distress Scale 22 (BDS-22)) anxiety (Lebanese Anxiety scale (LAS-10)) Insomnia (Lebanese Insomnia scale (LIS-18))	Fear of COVID-19 scale and financial well-being
Dataset 3	202	Knowledge, attitude, and practice toward COVID-19	Fear of COVID-19 scale
Dataset 4	2336	Having been diagnosed or not with COVID-19	Preventive measure scale
Dataset 5	324	Burnout scale (Maslach Burnout Inventory)	Soft skills and emotional intelligence
Dataset 6	405	Stigma discrimination scale (SDS-11)	Fear of COVID-19 scale, anxiety (LAS-10), and knowledge scale
Dataset 7	410	Eating behaviors (Eating disorder examination questionnaire (EDE))	Fear of COVID-19 scale, anxiety (LAS-10), and boredom (Short Boredom Proneness Scale)

### Data analysis

Data were analyzed using SPSS software version 25. Weighting was performed according to the number of inhabitants by age group and gender, as described by the latest official version of the Lebanese population estimates [15]. In descriptive statistics, means and standard deviations were considered for continuous variables and counts and percentages for categorical variables. Associations between dichotomous variables were calculated using OR, while beta coefficients served to assess associations between quantitative variables.

In each dataset, the relative difference between estimates was calculated to assess the gap between the sample and the population figures, measured by the absolute change between weighted and unweighted values in comparison to the unweighted value (Relative difference = (unweighted value–weighted value)/unweighted value). The function log base 10 (Log10) was used to stabilize variation within the values of the used variables with non-normal distribution. A further step in the analysis was to compare the correlation of the values of the variables in all datasets between weighted and unweighted methods using Pearson’s correlation coefficient. Multiple regressions were conducted, comparing weighted versus unweighted results from datasets primary data: multiple linear regressions when the dependent variable (DV) was continuous and logistic regressions when the DV was dichotomous.

Finally, multivariable regression analyses were conducted on secondary data to assess the effect of the gap in independent variables on the adjusted OR or beta coefficient (between the independent and dependent variables). In other words, this effect was assessed through the impact of the relative difference of age and gender on the relative change in adjusted OR or beta coefficient. The presence of a significant association between age, gender, and independent variable (IV) with the DV, using the weighted and unweighted methods in each dataset, was also taken into account. In all cases, a *p*-value < 0.05 was considered statistically significant.

## Results

### Description of age and gender using simple unweighted and weighted methods

Table 2 shows the distribution of age and gender in the seven datasets using simple unweighted and weighted methods. The proportions differed regarding age and gender. For example, in the first dataset, a high relative difference was mainly found in participants older than 45 (250%); a similar result was found in the third dataset for age < 35 years. Similarly, in the fifth dataset, a high relative difference was found between the two groups, essentially in those aged over 45 years (251.72%). In other subgroups, the relative difference could be as low as 3% in dataset 5 and 6.5% in dataset 6.

**Table 2:** Description of age and gender using simple (unweighted) and weighted methods

	**Weighted data** **(by age and gender)**	**Unweighted data (sample)**	**Relative difference percentage**
	**Frequency (%)**	**Frequency (%)**	
**Dataset 1**	***N* = 310**	***N* = 310**	
**Age**			
< 30 years	125 (40.2%)	171 (55.2%)	26.90
30 – 34 years	36 (11.5%)	54 (17.4%)	33.33
35 – 39 years	28 (9.2%)	32 (10.3%)	12.5
40 – 44 years	23 (7.5%)	25 (8.1%)	8.00
> 45 years	98 (31.6%)	28 (9.0%)	250
**Gender**			
Male	151 (48.6%)	95 (30.6%)	58.94
Female	159 (51.4%)	215 (69.4%)	26.04
**Dataset 2**	***N* = 508**	***N* = 509**	
**Age**			
< 30 years	204 (40.1%)	170 (33.4%)	20.00
30 – 34 years	59 (11.5%)	45 (8.8%)	31.11
35 – 39 years	47 (9.2%)	74 (14.5%)	36.48
40 – 44 years	38 (7.6%)	74 (14.5%)	48.64
> 45 years	161 (31.6%)	146 (28.7%)	10.27
**Gender**			
Male	248 (48.7%)	131 (25.7%)	89.31
Female	261 (51.3%)	378 (74.3%)	30.95
**Dataset 3**	***N* = 202**	***N* = 202**	
**Age**			
< 35 years	105 (51.8%)	30 (14.9%)	250
35 – 39 years	19 (9.2%)	14 (6.9%)	35.71
40 – 44 years	15 (7.5%)	31 (15.3%)	51.61
> 45 years	64 (31.6%)	127 (62.9%)	49.60
**Gender**			
Male	98 (48.6%)	122 (60.4%)	19.67
Female	104 (51.4%)	80 (39.6%)	30.0
**Dataset 4**	***N* = 2373**	***N* = 2336**	
**Age**			
< 30 years	944 (40.3%)	1111 (47.6%)	15.03
30 – 34 years	269 (11.5%)	264 (11.3%)	1.89
35 – 39 years	214 (9.2%)	304 (13.0%)	29.60
40 – 44 years	175 (7.5%)	251 (10.7%)	30.27
> 45 years	737 (31.5%)	406 (17.4%)	81.52
**Gender**			
Male	1144 (48.4%)	502 (21.3%)	127.88
Female	1218 (51.6%)	1856 (78.7%)	34.37
**Dataset 5**	***N* = 323**	***N* = 324**	
**Age**			
< 30 years	130 (40.1%)	203 (62.7%)	35.96
30 – 34 years	37 (11.6%)	30 (9.3%)	23.33
35 – 39 years	30 (9.2%)	31 (9.6%)	3.22
40 – 44 years	24 (7.5%)	31 (9.6%)	22.58
> 45 years	102 (31.6%)	29 (9.0%)	251.72
**Gender**			
Male	158 (48.8%)	64 (19.8%)	146.87
Female	165 (51.2%)	260 (80.2%)	36.53
**Dataset 6**	***N* = 405**	***N* = 405**	
**Age**			
< 20 years	57 (14.2%)	136 (33.6%)	58.08
20 – 24 years	56 (13.8%)	80 (19.8%)	30
25 – 29 years	49 (12.2%)	46 (11.4%)	6.52
30 – 34 years	47 (11.5%)	22 (5.4%)	113.63
> 35 years	196 (48.3%)	121 (29.9%)	61.98
**Gender**			
Male	197 (48.7%)	82 (20.2%)	140.24
Female	208 (51.3%)	323 (79.8%)	35.60
**Dataset 7**	***N* = 409**	***N* = 410**	
**Age**			
< 20 years	59 (14.3%)	36 (8.8%)	63.88
20 – 24 years	56 (13.8%)	105 (25.6%)	46.66
25 – 29 years	50 (12.1%)	104 (25.4%)	51.92
30 – 34 years	47 (11.6%)	63 (15.4%)	25.39
> 35 years	197 (48.2%)	102 (24.9%)	93.13
**Gender**			
Male	199 (48.7%)	79 (19.3%)	151.89
Female	210 (51.3%)	331 (80.7%)	36.55

### Description of variables using the weighted and unweighted methods

Table 3 summarizes the description of dependent variables (DV) and independent variables (IV) using simple (unweighted) and weighted methods. The weighting applied on demographic characteristics showed low relative differences, and the values were very similar between the two groups, whether variables were continuous or categorical. The bivariate analysis between the independent variables and the dependent variables are presented in the supplementary table 1.

**Table 3:** Description of the dependent and independent variables used in the databases

Sample survey			
	**Weighted data (by age and gender)**	**Unweighted data (sample)**	**Relative difference percentage**
**Dataset 1**	***N* = 310**	***N* = 310**	
Practice scale (DV)	10.73 ± 3.30	10.78 ± 3.27	0.46
Attitude scale (IV)	9.09 ± 3.80	9.20 ± 3.85	1.19
**Dataset 2**	***N* = 508**	***N* = 509**	
Stress scale (BDS-22) (DV)	16.77 ± 15.48	17.49 ± 15.65	4.11
Anxiety scale (LAS-10) (DV)	15.43 ± 8.78	15.75 ± 8.71	2.03
Insomnia scale (LIS-18) (DV)	44.92 ± 11.20	45.20 ± 11.28	0.61
Fear of COVID-19 scale (IV)	10.81 ± 5.91	11.08 ± 5.86	2.43
Financial well-being scale (IV)	39.70 ± 17.00	39.54 ± 17.29	0.40
**Dataset 3**	***N* = 202**	***N* = 202**	
Knowledge (DV)	25.43 ± 1.83	25.72 ± 1.79	1.12
Attitude (DV)	31.42 ± 4.69	31.31 ± 4.33	0.35
Practice (DV)	11.54 ± 0.90	11.48 ± 1.04	0.52
Fear of COVID-19 (IV)	15.80 ± 5.90	15.47 ± 5.78	2.13
**Dataset 4**	***N* = 2373**	***N* = 2336**	
Having been diagnosed or not with COVID-19			
Yes	514 (21.8%)	528 (22.4%)	2.65
No	1848 (78.2%)	1830 (77.6%)	0.98
Preventive measure scale (IV)	66.03 ± 9.81	66.07 ± 9.85	0.06
**Dataset 5**	***N* = 323**	***N* = 324**	
Burnout (DV)	58.59 ± 10.98	59.04 ± 11.61	0.76
Soft Skills (IV)	139.76 ± 18.46	138.59 ± 18.33	0.84
Emotional intelligence (IV)	85.31 ± 17.43	87.00 ± 14.99	1.94
**Dataset 6**	***N* = 405**	***N* = 405**	
Stigma discrimination scale (DV)	26.37 ± 4.91	26.25 ± 5.41	0.45
Fear of COVID-19 (IV)	17.55 ± 5.42	17.47 ± 5.53	0.45
Anxiety scale (LAS-10) (IV)	1.02 ± 2.39	1.16 ± 2.44	12.06
Knowledge scale (IV)	16.61 ± 2.59	16.29 ± 2.86	1.96
**Dataset 7**	***N* = 409**	***N* = 410**	
Score of eating behavior (DV)	1.36 ± 1.31	1.51 ± 1.38	9.93
Fear of COVID-19 (IV)	28.23 ± 9.13	29.16 ± 9.12	3.18
Anxiety scale (LAS-10) (IV)	13.66 ± 7.53	14.37 ± 7.88	4.94
Boredom proneness scale (IV)	23.41 ± 11.82	24.49 ± 12.20	4.40

^*^DV: dependent variable, IV: independent variable

### Correlation between unweighted and weighted values

**Fig. 1:**
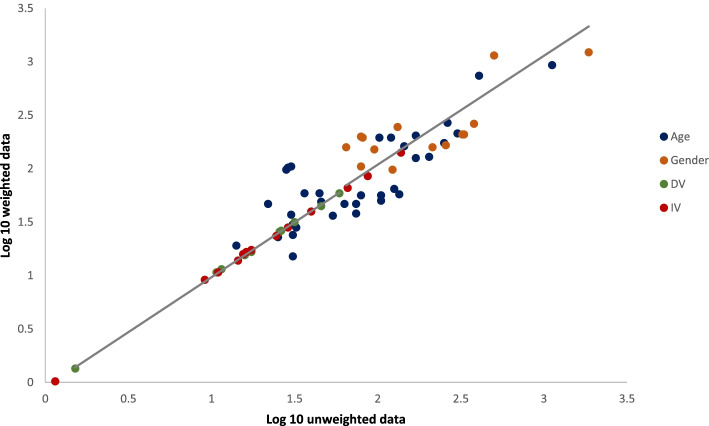
Correlation between weighted and unweighted data according to age, gender, dependent variables (DV), and independent variables (IV)

A strong positive correlation was found between the values of weighted and unweighted data taking into account the values of gender, age, dependent variables, and independent variables (*r* = 0.918, *p* < 0.001) (Fig. 1). Although lower than that of dependent variables (*r* = 1.000, *p* < 0.001), a positive correlation was found between unweighted and weighted values of age (*r* = 0.824, *p* < 0.001), gender (*r* = 0.780, *p* = 0.001), and independent variables (*r* = 1.000, *p* < 0.001).

### Correlation between relative differences of variables and association measure

A strong correlation was found between age relative difference (*r* = 0.863, *p* = 0.012) and the sample size (*r* = -0.891, *p* = 0.007) with the adjusted OR relative difference (Table 4). No significant association was found between the adjusted OR relative difference, gender, and the independent variable relative differences.

**Table 4:** Correlation between Relative differences

	**The adjusted OR relative difference**	***p*-value**
	**Correlation coefficient**	
Sample size	-0.891	**0.007**
Independent variable relative difference	0.309	0.500
Age relative difference	0.863	**0.012**
Gender relative difference	-0.559	0.192

### Multivariable analysis comparing weighted and unweighted samples

Table 5 displays the results of weighted and unweighted multivariable models (linear or logistic regressions), showing discrepancies between models.

**Table 5:** Multivariable analysis taking an outcome variable as the dependent variable

**Dataset 1: *n* = 310 Continuous dependent variable (Practice scale)**	**Unweighted linear regression**		**Linear regression weighted by age and gender**		**Relative difference**
	**Coefficient (B1)**	***p***	**Coefficient (B2)**	***p***	100*(B2-B1)/B1
Age	0.01 [-0.07,0.10]	0.79	0.008 [-0.06,0.08]	0.83	-20
Gender	0.33 [-0.49,1.16]	0.42	0.62 [-0.14,1.38]	0.11	87.87
Experience in community pharmacy (in years)	-0.01 [-0.12,0.10]	0.86	0.01 [-0.08,0.10]	0.82	200
Attitude scale	0.03 [-0.05,0.13]	0.44	0.07 [-0.02,0.17]	0.13	133.33
**Dataset 2: *n* = 509**	**Unweighted linear regression**		**Linear regression weighted by age and gender**		**Relative difference**
	**Coefficient (B1)**	***p***	**Coefficient (B2)**	***p***	100*(B2-B1)/B1
**Continuous dependent variable (BDS-22)**					
Age	-0.29 [-0.38,-0.19]	** < 0.001**	-0.28 [-0.37,-0.19]	** < 0.001**	3.44
Gender	2.81 [0.07,5.56]	**0.040**	2.97 [0.61,5.32]	**0.010**	5.69
Fear of COVID-19	1.05 [0.84,1.26]	** < 0.001**	1.06 [0.85,1.27]	** < 0.001**	0.95
Financial well-being scale	-0.11 [-0.18,-0.04]	**0.001**	-0.12 [-0.19,-0.04]	**0.001**	-9.09
**Continuous dependent variable (LAS-10)**					
Age	-0.09 [-0.15,-0.03]	**0.002**	-0.09 [-0.14,-0.03]	**0.001**	0
Gender	1.14 [-0.50,2.78]	0.17	1.21[-0.24,2.67]	0.10	6.14
Fear of COVID-19	0.41 [0.28,0.54]	** < 0.001**	0.43 [0.30,0.56]	** < 0.001**	4.87
Financial wellbeing scale	-0.50 [-0.09,-0.007]	**0.02**	-0.01 [-0.06,0.02]	0.38	-98
**Continuous dependent variable (LIS-18)**					
Age	-0.02 [-0.09,0.05]	0.54	0.01 [-0.05,0.08]	0.63	150
Gender	1.38 [-0.71,3.49]	0.19	1.22 [-0.61,3.06]	0.19	-11.59
Fear of COVID-19	0.47 [0.31,0.63]	** < 0.001**	0.45 [0.29,0.61]	** < 0.001**	-4.25
Financial well-being scale	-0.13 [-0.19,-0.08]	** < 0.001**	-0.13 [-0.18,-0.07]	** < 0.001**	0
**Dataset 3: n = 202**	**Unweighted linear regression**		**Linear regression weighted by age and gender**		**Relative difference**
	**Coefficient (B1)**	***p***	**Coefficient(B2)**	***p***	100*(B2-B1)/B1
**Continuous dependent variable (Knowledge)**					
Age	0.005 [-0.01,0.02]	0.65	0.02 [-0.001,0.04]	0.06	300
Gender	0.41 [-0.11,0.95]	0.12	0.53 [0.03,1.03]	**0.03**	29.26
Major independent variable (Fear of COVID-19)	-0.02 [-0.07,0.01]	0.18	-0.05 [-0.09,-0.01]	**0.01**	-150
**Continuous dependent variable (Attitude)**					
Age	-0.01 [-0.07,0.03]	0.49	-0.02 [-0.07,0.03]	0.50	-100
Gender	-0.96 [-2.25,0.33]	0.14	-0.97 [-2.28,0.34]	0.14	-1.04
Major independent variable (Fear of COVID-19)	-0.008 [-0.11,0.09]	0.88	0.02 [-0.08,0.13]	0.68	350
**Continuous dependent variable (Practice)**					
Age	0.002 [-0.01,0.01]	0.77	-0.002 [-0.01,0.009]	0.67	-200
Gender	0.20 [-0.10,0.51]	0.19	0.26 [0.01,0.51]	**0.03**	30
Major independent variable (Fear of COVID-19)	0.003 [-0.02,0.02]	0.80	0.03 [0.01,0.05]	**0.004**	900
**Dataset 4: *n* = 2336** **Dichotomous dependent variable (Having been diagnosed or not with COVID-19)**	**Unweighted logistic regression**		**Logistic regression weighted by age and gender**		**Relative difference**
	**OR1**	***p***	**OR2**	***p***	100*(OR2-OR1)/OR1
Age	0.99 [0.98,1.00]	0.38	0.99 [0.98,1.00]	0.07	0
Gender	1.00 [0.78,1.27]	0.99	0.99 [0.81,1.21]	0.95	-1
Major independent variable (Preventive measure scale)	1.00 [0.99,1.01]	0.19	1.01 [1.00,1.02]	**0.002**	1
**Dataset 5: *n* = 324** **Continuous dependent variable (Burnout)**	**Unweighted linear regression**		**Linear regression weighted by age and gender**		**Relative difference**
	**Coefficient (B1)**	***p***	**Coefficient (B2)**	***p***	100*(B2-B1)/B1
Age	-0.008 [-0.15,0.13]	0.91	0.007 [-0.08,0.10]	0.88	187.5
Gender	2.71 [-0.36,5.79]	0.08	2.59 [0.21,4.97]	**0.03**	-4.42
Major independent variable (Soft Skills)	-0.15 [-0.23,-0.07]	** < 0.001**	-0.10 [-0.18,-0.03]	**0.005**	-33.33
Other independent variable (Emotional intelligence)	-0.04 [-0.14,0.04]	0.30	-0.08 [-0.16,0.001]	0.051	-100
**Dataset 6: *n* = 405** **Continuous dependent variable (Stigma scale)**	**Unweighted linear regression**		**Linear regression weighted by age and gender**		**Relative difference**
	**Coefficient (B1)**	***p***	**Coefficient (B2)**	***p***	100*(B2-B1)/B1
Age	0.009 [-0.03,0.04]	0.65	0.04 [0.007,0.07]	**0.02**	344.44
Gender	-0.30 [-1.52,0.88]	0.61	-0.55 [-1.45,0.34]	0.22	-83.33
Fear of COVID-19	-0.04 [-0.14,0.05]	0.36	-0.02 [-0.11,0.07]	0.67	50
Anxiety	0.09 [-0.12,0.31]	0.39	0.07 [-0.13,0.28]	0.48	-22.22
Knowledge scale	-0.33 [-0.48,-0.15]	** < 0.001**	-0.31 [-0.49,-0.14]	** < 0.001**	6.06
**Dataset 7: *n* = 410** **Continuous dependent variable (Total score of eating behavior)**	**Unweighted linear regression**		**Linear regression weighted by age and gender**		**Relative difference**
	**Coefficient (B1)**	***P***	**Coefficient (B2)**	***p***	100*(B2-B1)/B1
Age	-0.006 [-0.02,0.008]	0.38	-0.009 [-0.02,0.002]	0.10	-50
Gender	0.35 [0.02,0.67]	**0.03**	0.38 [0.14,0.62]	**0.002**	8.57
Fear of COVID-19	0.01 [0.002,0.03]	**0.02**	0.02 [0.01,0.03]	**0.001**	100
Anxiety	0.03 [0.01,0.05]	** < 0.001**	0.03 [0.01,0.05]	**0.001**	0
Boredom scale	0.007 [-0.006,0.02]	0.29	0.005 [-0.008,0.01]	0.46	-28.57

In the first dataset (*N* = 310), the association of the independent variable (attitude toward COVID-19) with the dependent variable (practice toward COVID-19) remained not significant (*p*-value > 0.05) between the two methods used. However, there was an increase in the relative difference by 133.33% between unweighted and weighted values.

In the second dataset (*N* = 509), the association of the independent variables (fear of COVID-19 and financial well-being) with the dependent variables (stress, anxiety, and insomnia) remained significant in both methods when considering the three dependent variables, except for the model where the dependent variable was anxiety (LAS-10). In the latter, the financial well-being scale (IV) yielded a significant association in the unweighted regression (*p* = 0.02) but a non-significant result in the weighted regression (*p* = 0.38). The weighted beta value was 98% lower than the unweighted beta value.

In the third dataset (*N* = 202), the association of the independent variable (fear of COVID-19) with the dependent variables (knowledge and practice) was not significant in the unweighted sample. However, a statistically significant association was found in the weighted sample. A relative increase in beta value was found for gender in the weighted method, with a beta decrease of 150% for the independent variable. When considering the attitude scale as the dependent variable, no significant association was found between the IV and the DV using the two methods.

In the fourth dataset (*N* = 2336), the association of the independent variable (preventive measure scale) with the dependent variable (having been diagnosed or not with COVID-19) was not significant in the unweighted sample. However, a statistically significant association was found in the weighted sample. Relative differences in OR varied between -1% and 1% after weighting.

In the fifth dataset (*N* = 324), the association of the independent variables (soft skills and emotional intelligence) with the dependent variable (burnout scale) yielded different results. It was significant for soft skills in both methods, while emotional intelligence remained non-significant when using the two methods, with a p-value tending to be significant in the weighted sample. A negative relative difference was found for the independent variable after weighting.

In the sixth dataset (*N* = 405), the association of the independent variable (knowledge scale) with the dependent variable (stigma discrimination scale) was significant in both methods. The fear of COVID-19 and anxiety remained non-significant when using the two methods. A decrease or increase in the relative difference was found after weighting.

In the seventh dataset (*N* = 410), the association of the major independent variables (fear of COVID-19 and anxiety) with the dependent variable (eating behaviors) was significant in both methods. The boredom scale remained non-significant when using the two methods. Relative differences varied after weighting.

### Secondary data analysis: factors affecting the relative change of major association measures

Table 6 displays the association between age, gender, independent variable gaps (between sample and population), associations significance, and the sample size with the major association relative change. The results showed that a larger sample size (Beta = -0.001, *p* = 0.001), a higher gender gap (Beta = -0.007, *p* = 0.003), and the presence of a significant association between weighted age and the DV (Beta = -0.221, *p* = 0.013) would significantly decrease the relative change of the major association. However, a higher age gap (Beta = 0.010, *p* = 0.005) was significantly associated with a higher relative change in the major association. In terms of absolute impact, the highest impact on the association measure was related to sample size, followed by age relative difference, gender relative difference, and finally, the significance of the association between weighted age and the dependent variable.

**Table 6:** Linear regression taking the relative change in the major association as the dependent variable

	**Standardized beta**	**Unstandardized beta**	**95% CI** **LL; UL**	***p*-value**
Sample size	-0.686	-0.001	-0.0008, -0.0006	0.001
Age relative difference	0.284	0.010	0.007, 0.013	0.005
Gender relative difference	-0.216	-0.007	-0.008, -0.005	0.003
Significant association for weighted age with the DV (Yes vs. No*)	-0.129	-0.221	-0.331, -0.110	0.013

*CI* Confidence interval, *LL* Lower level, *UL* Upper level

## Discussion

Our study compared weighted and unweighted samples of online surveys and assessed the extent to which weighting methods can adjust the web sampling to the reference sample and how it would affect the results. Our findings revealed a high variation of age and gender between weighted and unweighted samples within the same population; however, high similarities were found for dependent and independent variables in terms of relative difference measures.

The regression analysis results showed a high relative difference between weighting and unweighting methods in some datasets and for some variables, while a low difference was found for others; association measures would increase or decrease after weighting. These discrepancies could be explained by the large sample size and the high relative difference in gender, related to lower relative differences in association measures between weighted and unweighted methods. However, a high relative difference of age was associated with the high relative difference of association measure. These results indicate that proportions of the sociodemographic variables are adjusted after applying the weighting methods; however, it does not necessarily affect the association between variables.

The impact of weighting was limited in some datasets, while differences were found in others. The discrepancies between weighted and unweighted databases were significantly affected by the sample size, followed by age relative difference and gender relative difference. A possible explanation could be that when analyzing the use of weights to compensate for the distributions of different variables, some factors of interest may not always have a strong enough link with the demographic variables; thus, the weighting method could not correct any biases. Consequently, the impact of weighting depends on the variables of interest and how these variables are related to the sociodemographic variables. As a result, the decision to weight samples will be based on the study objective, design, and type of outcome.

Our work showed that the initial gap between the sample and the population, in addition to the sample size and the presence of a significant association between some sociodemographic variables and the dependent variable, could all impact the association measure, but in differential ways: correcting for age gap would improve association measures, but not gender gap correction. Similarly, other researchers had previously reported that weighting techniques can compensate for proportionality but not always representativeness because some factors of interest do not always have a strong enough link with the demographic weighting variables [16]. Thus, adjusting for proportionate overrepresentation and underrepresentation of specific respondent categories does not imply that the substantive responses of online access panel respondents are equal to those of the general population [16]. Oppositely, according to Bethlehem and Stoop, one or more qualitative auxiliary variables are required for the weighting method. Nevertheless, even if the target variable and the stratification variables have a strong relationship, the change in the target variable's values appears very low [16].

Our results showed that the larger the sample size, the lower the impact on the association measure; in other words, lower samples derived association measures are more affected if not weighted. This finding corroborates the principle that large sample sizes and high response rates positively influence the quality of estimates, according to the theoretical framework of probability sampling [17]. Similarly, a large-scale study that used 17 samples from online surveys found that bigger sample size (lower margin of sampling error around the estimate) is associated with a better level of precision [12]; large-scale online surveys have the advantage that specific subgroups can be identified [16]. The fundamental assumption is that people who engage in an online survey, whether elderly single women, less educated people, ethnic minorities, or other usually underrepresented groups, are equivalent to those who do not engage in online surveys [16], even though people who belong to these groups are hard to reach or unlikely to participate in surveys [16]. However, one study has addressed the erroneous idea that larger samples imply more valid replies [18], showing that larger samples do not always yield better estimates than smaller ones from non-probability samples, while a larger sample size can lead to greater accuracy only with probability samples [18]. Similarly, according to Bryman and Bell, precision cannot be guaranteed with a large sample size [19]. Thus, additional studies are required to further depict these findings.

Our findings reinforce the variability of results found in the literature about the application of weighting methods in scientific surveys. It is unclear whether or not online surveys can be made more representative [20]. While weighted samples are expected to be more representative than unweighted ones, this study could demonstrate that this is not always the case. As a result, one cannot simply assume that using the weighted method will always result in a more accurate estimate of the population studied. Also, it cannot be concluded that the unweighted technique will always yield more conservative sample homogeneity suggestions than the sample-weighted method, as demonstrated by previous findings showing that demographic weighting reduced bias in some cases and increased it in others [11, 12]. A study compared data from a self-administered online survey with the answers collected in a face-to-face interview and found that the results were not significantly affected by weights on age, gender, or education [8]. Another study compared two datasets collected online and showed that the impact of the weighting method on the results was very limited [1]. Other findings revealed that non-probability samples significantly differed from probability samples, particularly in terms of attitudes and behaviors, even after making them demographically similar to target groups [11, 21–23].

### Limitations

This study has several limitations. The online samples were not compared to face-to-face interviews, which could have presented more reasonable results. The sampling selectivity and the inconsistency of variables used on each survey may have affected the results. Conclusions comparing inequities in weighted and unweighted populations may change depending on the variable of interest. Variables other than demographics were not taken into account for adjustment, which could also affect the results. Different weighting techniques, such as the propensity score technique, were not applied.

## Conclusion

The results of this analysis of online surveys indicate that weighting methods should be used cautiously, as weighting did not affect the results in some datasets, while it did in others. Weighting methods might yield unpredictable results, depending on variable gaps, sample size, and the association between sociodemographic characteristics used for adjustment and dependent variables. Further research is necessary to define situations in which weighting would be beneficial.

## Supplementary Information

**Additional file 1.**

## Data Availability

The datasets used and/or analyzed during the current study are available from the corresponding author upon reasonable request.

## Abbreviations

COVID-19: Coronavirus disease 2019
BDS-22: Beirut Distress Scale
LAS-10: Lebanese Anxiety Scale
LIS-18: Lebanese Insomnia Scale
SDS-11: Stigma Discrimination Scale
EDE: Eating Disorder Examination questionnaire
SPSS: Statistical Package for the Social Sciences
DV: Dependent variable
IV: Independent variable
OR: Odds Ratio
