# Supplementary material for: Should samples be weighted to decrease selection bias in online surveys during the COVID-19 pandemic? Data from seven datasets
Source: BMC Med Res Methodol. 2022 Mar 6;22:63. doi: 10.1186/s12874-022-01547-3 (PMC8898325; doi:10.1186/s12874-022-01547-3)
Supplement: Supplementary file 1 — Additional file 1. [file 12874_2022_1547_MOESM1_ESM.docx]

**Supplementary material**

| **Supplementary Table 1: Bivariate analysis** | | | | | | |
| --- | --- | --- | --- | --- | --- | --- |
|  | **Weighted data**  **(by age and gender)** | | | **Unweighted data (sample)** | | |
| **Dataset 1 (n=310)** | **DV: Practice scale** | | **p-value** | **DV: Practice scale** | | **p-value** |
|  | **Correlation coefficient** | |  | **Correlation coefficient** | |  |
| **Attitude scale (IV)** | 0.088 | | 0.123 | 0.044 | | 0.435 |
| **Experience in community pharmacy (years)** | 0.030 | | 0.602 | -0.007 | | 0.908 |
| **Age** | 0.041 | | 0.473 | 0.004 | | 0.950 |
| **Gender** | **Mean ± SD** | |  | **Mean ± SD** | |  |
| Male | 10.41 ± 3.45 | | 0.094 | 10.52 ± 3.35 | | 0.352 |
| Female | 11.04 ± 3.14 | |  | 10.90 ± 3.24 | |  |
| **Dataset 2 (N=508)** | **DV: Stress scale (BDS-22)** | | **p-value** | **DV: Stress scale (BDS-22** | | **p-value** |
|  | **Correlation coefficient** | |  | **Correlation coefficient** | |  |
| **Fear of COVID-19 scale (IV)** | 0.397 | | **<0.001** | 0.395 | | **<0.001** |
| **Financial well-being scale (IV)** | -0.217 | | **<0.001** | -0.221 | | **<0.001** |
| **Age** | -0.176 | | **<0.001** | -0.178 | | **<0.001** |
| **Gender** | **Mean ± SD** | |  | **Mean ± SD** | |  |
| Male | 14.78 ± 14.69 | | **0.005** | 14.67 ± 14.79 | | **0.017** |
| Female | 18.67 ± 15.98 | |  | 18.46 ± 15.84 | |  |
|  | **DV: Anxiety scale (LAS-10)** | | **p-value** | **DV: Anxiety scale (LAS-10)** | | **p-value** |
|  | **Correlation coefficient** | |  | **Correlation coefficient** | |  |
| **Fear of COVID-19 scale (IV)** | 0.280 | | **<0.001** | 0.284 | | **<0.001** |
| **Financial well-being scale (IV)** | -0.099 | | **0.025** | -0.163 | | **<0.001** |
| **Age** | -0.093 | | **0.035** | -0.093 | | **0.035** |
| **Gender** | **Mean ± SD** | |  | **Mean ± SD** | |  |
| Male | 14.63 ± 8.86 | | **0.046** | 14.63 ± 8.91 | | 0.087 |
| Female | 16.19 ± 8.65 | |  | 16.14 ± 8.62 | |  |
|  | **DV: Insomnia scale (LIS-18)** | | **p-value** | **DV: Insomnia scale (LIS-18)** | | **p-value** |
|  | **Correlation coefficient** | |  | **Correlation coefficient** | |  |
| **Fear of COVID-19 scale (IV)** | 0.290 | | **<0.001** | 0.296 | | **<0.001** |
| **Financial well-being scale (IV)** | -0.252 | | **<0.001** | -0.271 | | **<0.001** |
| **Age** | 0.063 | | 0.153 | 0.010 | | 0.817 |
| **Gender** | **Mean ± SD** | |  | **Mean ± SD** | |  |
| Male | 44.07 ± 11.25 | | 0.094 | 43.83 ± 11.19 | | 0.109 |
| Female | 45.73 ± 11.12 | |  | 45.67 ± 11.29 | |  |
| **Dataset 3 (N=202)** | **Knowledge (DV)** | | **p-value** | **Knowledge (DV)** | | **p-value** |
|  | **Correlation coefficient** | |  | **Correlation coefficient** | |  |
| **Fear of COVID-19 (IV)** | -0.184 | | **0.009** | -0.096 | | 0.172 |
| **Age** | 0.121 | | 0.086 | -0.004 | | 0.956 |
| **Gender** | **Mean ± SD** | |  | **Mean ± SD** | |  |
| Male | 25.16 ± 1.99 | | **0.041** | 25.56 ± 1.83 | | 0.125 |
| Female | 25.69 ± 1.64 | |  | 25.96 ± 1.71 | |  |
|  | **Attitude (DV)** | | **p-value** | **Attitude (DV)** | | **p-value** |
|  | **Correlation coefficient** | |  | **Correlation coefficient** | |  |
| **Fear of COVID-19 (IV)** | 0.036 | | 0.615 | -0.008 | | 0.915 |
| **Age** | -0.040 | | 0.573 | -0.018 | | 0.794 |
| **Gender** | **Mean ± SD** | |  | **Mean ± SD** | |  |
| Male | 31.90 ± 3.65 | | 0.153 | 31.63 ± 4.22 | | 0.186 |
| Female | 30.96 ± 5.47 | |  | 30.81 ± 4.49 | |  |
|  | **Practice (DV)** | | **p-value** | **Practice (DV)** | | **p-value** |
|  | **Correlation coefficient** | |  | **Correlation coefficient** | |  |
| **Fear of COVID-19 (IV)** | 0.192 | | **0.006** | 0.014 | | 0.839 |
| **Age** | -0.046 | | 0.513 | -0.006 | | 0.929 |
| **Gender** | **Mean ± SD** | |  | **Mean ± SD** | |  |
| Male | 11.41 ± 1.00 | | 0.050 | 11.40 ± 1.11 | | 0.206 |
| Female | 11.67 ± 0.78 | |  | 11.60 ± 0.92 | |  |
| **Dataset 4 (N=2373)** | **Having been diagnosed or not with COVID-19** | | **p-value** | **Having been diagnosed or not with COVID-19** | | **p-value** |
|  | **Yes** | **No** |  | **Yes** | **No** |  |
|  | **Mean ± SD** | **Mean ± SD** |  | **Mean ± SD** | **Mean ± SD** |  |
| **Preventive measure scale (IV)** | 67.22± 8.85 | 65.70±10.03 | **0.001** | 66.53±9.03 | 65.93±10.07 | 0.194 |
| **Age** | 35.90 ± 12.54 | 37.13 ± 13.76 | 0.056 | 33.28±10.69 | 33.79±11.72 | 0.345 |
| **Gender** | **Frequency (%)** | **Frequency (%)** |  | **Frequency (%)** | **Frequency (%)** |  |
| Male | 249 (21.7%) | 896 (78.3%) | 0.957 | 112 (22.3%) | 390 (77.7%) | 0.961 |
| Female | 266 (21.8%) | 952 (78.2%) |  | 416 (22.4%) | 1440 (77.6%) |  |
| **Dataset 5 (N=232)** | **Burnout (DV)** | | **p-value** | **Burnout (DV)** | | **p-value** |
|  | **Correlation coefficient** | |  | **Correlation coefficient** | |  |
| **Soft Skills (IV)** | -0.244 | | **<0.001** | -0.279 | | **<0.001** |
| **Emotional intelligence (IV)** | -0.201 | | **<0.001** | -0.169 | | **0.002** |
| **Age** | 0.042 | | 0.450 | -0.041 | | 0.465 |
| **Gender** | **Mean ± SD** | |  | **Mean ± SD** | |  |
| Male | 57.57 ± 10.47 | | 0.106 | 56.51 ± 12.11 | | 0.052 |
| Female | 59.55 ± 11.38 | |  | 59.66 ± 11.43 | |  |
| **Dataset 6 (N=405)** | **Stigma discrimination scale (DV)** | | **p-value** | **Stigma discrimination scale (DV)** | | **p-value** |
|  | **Correlation coefficient** | |  | **Correlation coefficient** | |  |
| **Fear of COVID-19 (IV)** | 0.147 | | **0.003** | 0.127 | | **0.010** |
| **Anxiety scale (LAS-10) (IV)** | 0.081 | | 0.104 | 0.118 | | **0.017** |
| **Knowledge scale (IV)** | -0.063 | | 0.203 | -0.109 | | **0.028** |
| **Age** | 0.200 | | **<0.001** | 0.066 | | 0.183 |
| **Gender** | **Mean ± SD** | |  | **Mean ± SD** | |  |
| Male | 26.88 ± 4.66 | | **0.039** | 26.96 ± 5.10 | | 0.185 |
| Female | 25.88 ± 5.11 | |  | 26.07 ± 5.48 | |  |
| **Dataset 7 (N=409)** | **Score of eating behavior (DV)** | | **p-value** | **Score of eating behavior (DV)** | | **p-value** |
|  | **Correlation coefficient** | |  | **Correlation coefficient** | |  |
| **Fear of COVID-19 (IV)** | 0.273 | | **<0.001** | 0.226 | | **<0.001** |
| **Anxiety scale (LAS-10) (IV)** | 0.276 | | **<0.001** | 0.300 | | **<0.001** |
| **Boredom proneness scale (IV)** | 0.215 | | **<0.001** | 0.226 | | **<0.001** |
| **Age** | -0.153 | | **0.002** | -0.110 | | **0.025** |
| **Gender** | **Mean ± SD** | |  | **Mean ± SD** | |  |
| Male | 1.13 ± 1.11 | | **0.001** | 1.13 ± 1.07 | | **0.002** |
| Female | 1.57 ± 1.44 | |  | 1.59 ± 1.43 | |  |
| *Values marked in bold are significant | | | | | | |
